# Asymmetric synthesis of a high added value chiral amine using immobilized ω-transaminases

**DOI:** 10.3762/bjoc.15.6

**Published:** 2019-01-07

**Authors:** Antonella Petri, Valeria Colonna, Oreste Piccolo

**Affiliations:** 1 Dipartimento di Chimica e Chimica Industriale, Università di Pisa, Via Giuseppe Moruzzi 13, 56124 Pisa, Italy; 2 Studio di Consulenza Scientifica (SCSOP), Via Bornò 5, 23896 Sirtori (LC), Italy

**Keywords:** asymmetric catalysis, biotransformations, chiral amine, immobilized enzymes, ω-transaminases

## Abstract

Chiral N-heterocyclic molecules and in particular compounds with an amino functional group such as 3-aminopiperidine are valuable intermediates for the production of a large number of bioactive compounds with pharmacological properties. In this paper, the synthesis of both enantiomers of 3-amino-1-Boc-piperidine by amination of the prochiral precursor 1-Boc-3-piperidone using immobilized ω-transaminases (TAs-IMB), isopropylamine as amine donor and pyridoxal-5’-phosphate (PLP) as cofactor is described. Compared to other methods, the present approach affords the target compound in just one step with high yield and high enantiomeric excess starting from a commercial substrate. The reaction was carried out by using different commercially available immobilized enzymes, evaluating the catalytic activity and the enantioselectivity under different experimental conditions. Re-use of the most efficient enzyme was performed both in batch and in a semi-continuous system. The selected biocatalyst showed good stability under the reaction conditions providing consistent results in terms of conversion and enantiomeric excess after several cycles. The reported results may be of practical interest in view of the development of this sustainable approach to an industrial scale.

## Introduction

Enantiomerically pure amines are important precursors to biologically active compounds with different industrial applications, including pharmaceuticals, fragrances and agricultural products [1]. It is therefore important to develop methods for their preparation which can be suitable for a large scale production. In this context there is an increasing interest in reactions including ω-transaminases (TAs) which have been identified as a greener and more sustainable method for chiral amine production [2–5]. TAs, also known as aminotransferases, are enzymes capable of transferring an amino group from an amine donor to an acceptor containing a carbonyl functionality in the presence of pyridoxal-5'-phosphate (PLP) as a cofactor and the enzymes are easily regenerated in situ without the need for another enzyme. In principle, enantiomerically pure chiral amines can be prepared following two approaches: through kinetic resolution starting from racemic amines or by asymmetric synthesis starting from suitable substrates, e.g., the corresponding carbonyl compounds. In a kinetic resolution, a maximum yield of 50% of the product can be obtained. Moreover, high quantities of the co-product might complicate the product separation and the recovery of the chiral amine. Thus, the asymmetric synthesis is generally preferable because a theoretically 100% yield of the product is possible. However, this process is reversible and therefore the conversion of the substrate is determined by how much the equilibrium is shifted towards the formation of the desired product. For this purpose two different strategies are used: (i) removal of the co-product deriving from the amine donor (i.e., under reduced pressure or by degradation/transformation) and (ii) use of an excess of the amine donor. The latter method might have, however, some drawbacks: the recovery and purification of the target amine can be difficult depending on the nature of the amine donor used in excess and/or this excess can cause inhibition of the enzyme. The use of isopropylamine (IPA) as amino donor allowed optimizing the use of TAs in organic synthesis thanks to its ability to shift the transamination reactions towards complete conversion together with easy removal of the low boiling point byproduct acetone [2–6].

Since (*R*)- and (*S*)-selective TAs are available on the market, both enantiomers of the amine product are accessible. This point is particularly relevant since in most cases the pharmacological activity of a chiral drug is closely related to its absolute configuration. Recent studies have favored the application of TAs at the industrial level, so it is not surprising that they are frequently found among the enzymes designed for the large-scale synthesis of chiral amines [2,4]. Moreover, immobilization of TAs has been developed in order to increase stability, enzyme recyclability, easy work-up and purification of the product [2–4,7–13]. Recently, we have reported a successful use of several immobilized TAs with IPA as amine donor [14].

Optically active 3-aminopiperidines and their N and/or N'-protected analogues, are important intermediates for the synthesis of a large number of biologically active compounds used for the treatment of obesity, type-I and II diabetes mellitus or as psychotropic drugs against depression and schizophrenia [15–19]. In particular (*R*)-3-amino-1-Boc-piperidine is a useful precursor of compounds mainly used for the synthesis of dipeptidyl peptidase IV (DPP-IV) inhibitors, such as alogliptin, linagliptin and other antidiabetic agents [15,17]. Over the years, numerous synthetic pathways were tested for the preparation of 3-aminopiperidine and its N-protected precursors in an optically active form by using resolution of racemic mixtures, preparation from chiral precursors or asymmetric synthesis from prochiral compounds [20–22].

In contrast, only few examples have been published on the synthesis of this molecule through biotransformations [23–24]. In these procedures TAs are used in commercial free form or isolated from bacterial sources, both in kinetic resolution starting from the corresponding racemic amine and in asymmetric synthesis starting from the corresponding ketone. The first method, although it proceeds with high enantioselectivity, is a kinetic resolution and as a consequence is limited to 50% maximum yield of the desired product. The second process employs non-commercial enzymes which might be a limitation in the perspective of a potential large-scale industrial application. More recently, the synthesis of the (*S*)-enantiomer of minor application interest has been described [25]. However, the enzyme used in this study is not commercially available and no enantioselectivity of the reaction has been reported.

Herein, we describe the use of several immobilized ω-transaminases in the asymmetric transamination of the selected substrate 1-Boc-3-piperidone. Selected reaction parameters were studied to optimize the protocol for further scaling-up purposes.

## Results and Discussion

**Scheme 1:**
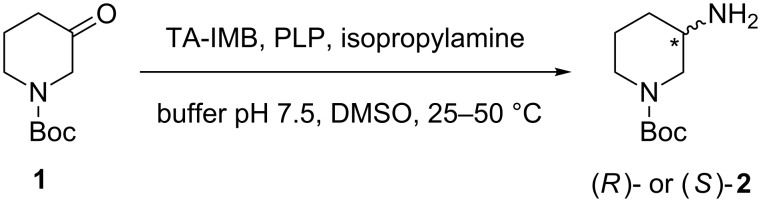
Transamination reaction of 1-Boc-3-piperidone (**1**).

In order to obtain enantiomerically pure (*R*)- and (*S*)-3-amino-1-Boc-piperidine (**2**), the transamination reaction was studied by using commercially available immobilized TAs (TAs-IMB) and isopropylamine as the amine donor (Scheme 1).

These enzymes are covalently immobilized on an epoxyacrylate resin and offer several advantages over the native ones, especially in view of a large scale application. In addition to an enhanced stability at higher temperatures or in organic solvents, immobilization on the support allows an easy recovery of the enzyme and isolation of the product after the reaction. Moreover, the activity of immobilized TAs used at appropriate concentrations can be comparable to that of the free enzymes [26]. The conditions for the application of these enzymes according to the supplier protocol are: 50 mg of substrate (45 mM), 200 mg of TA enzyme, 1 mM PLP, and 1 mM isopropylamine in triethanolamine buffer (100 mM, pH 7.5) in a total volume of 5.75 mL. If the substrate is insoluble in the buffer, a DMSO solution (15% v/v) can be used. These conditions were taken as a starting point to evaluate the reactivity and stereoselectivity of the enzymes in the transamination reaction of substrate **1** and the results are reported in Table 1.

**Table 1:** Screening of TAs-IMB for transamination reaction of **1**.

entry^a^	enzyme	time (h)	yield (%)^b^	ee (%)^c^

1	ATA-025-IMB	21	99	>99 (*R*)
2	ATA-013-IMB	52	65	>99 (*R*)
3	ATA-415-IMB	70	81	>99 (*R*)
4	ATA-303-IMB	360	90	>99 (*R*)
5	ATA-P1-G05-IMB	313	99	98 (*S*)
6	ATA-260-IMB	336	80	>99 (*S*)
7	ATA-256-IMB	336	80	>99 (*S*)
8	ATA-254-IMB	336	94	>99 (*S*)
9	ATA-301-IMB	360	90	n.d.^d^
10	ATA-234-IMB	360	97	n.d.^d^

^a^Reaction conditions: 50 mg of substrate (9 g/L, 45 mM, DMSO solution), 200 mg of enzyme, 550 rpm, 25 °C. ^b^Determined by HPLC analysis. ^c^Determined by HPLC analysis after derivatization. ^d^n.d.: not determined due to the presence of several byproducts.

As can be seen in Table 1, ATA-025-IMB was the most efficient among the (*R*)-selective enzymes since it led to high conversion and enantiomeric excess in the shortest reaction time (Table 1, entry 1). Accordingly, ATA-P1-G05-IMB was the most efficient in order to obtain the (*S*)-enantiomer (Table 1, entry 5).

In contrast, the (*S*)-selective enzymes ATA-260-IMB, ATA-256-IMB and ATA-254 allowed for the formation of the product with high enantioselectivity (>99%) but longer reaction times were required to achieve high yields of up to 94% (Table 1, entries 6, 7, and 8).

It should be noted that the TAs-IMB enzymes showed excellent enantioselectivities as demonstrated by the enantiomeric excess values which were all above 98%. Therefore, these results represent an improvement in the preparation of enantiomerically pure **2** compared with literature data [23]. Höhne et al. obtained only the (*R*)-enantiomer in 42% yield and 97% ee as a result of kinetic resolution of **1** using ω-transaminase and pyruvate as an amine acceptor. The enantiomeric excess in entries 9 and 10 of Table 1 was not determined due to the presence of several byproducts.

Transamination reactions using these optimized reaction conditions with commercially available TAs-IMB have been performed with other achiral [14] and pro-chiral [27] substrates, such as for example 4-methoxyphenylacetone. It was found that quantitative conversions were in some cases obtained only with (*S*)-selective TAs, confirming that it is difficult to predict the reactivity of the enzymes by changing the nature of the substrate.

After these initial investigations at room temperature, four promising enzymes were selected (i.e., three (*R*)-selective and one (*S*)-selective) which allowed to obtain high conversions and enantiomeric excesses in the transamination reaction of **1**.

In order to enhance reaction rates, an optimization study was undertaken within the stability range of the enzyme. According to the supplier, the used TAs-IMB showed a higher stability in the range between 30 °C and 55 °C when the model compound acetophenone was used as substrate. We were interested in exploring the behavior of these enzymes using **1** as starting material. Transamination reactions catalyzed by ATA-025-IMB, ATA-415-IMB, ATA-013-IMB, and ATA-P1-G05-IMB were repeated under the same experimental conditions described above with the exception of a higher reaction temperature. The results are shown in Table 2.

**Table 2:** Investigation of the temperature effect on the transamination reaction of **1**.

entry^a^	enzyme	temperature (°C)	time (h)	yield (%)^b^	ee (%)^c^

1	ATA-025-IMB	35	5	99	>99 (*R*)
2	ATA-025-IMB	50	3	99	>99 (*R*)
3	ATA-415-IMB	35	26	99	>99 (*R*)
4	ATA-415-IMB	50	24	99	>99 (*R*)
5	ATA-013-IMB	35	51	93	>99 (*R*)
6	ATA-013-IMB	50	28	99	>99 (*R*)
7	ATA-P1-G05-IMB	35	75	92	98 (*S*)
8	ATA-P1-G05-IMB	50	50	99	98 (*S*)

^a^Reaction conditions: 50 mg of substrate (9 g/L, 45 mM, DMSO solution), 200 mg of enzyme, 550 rpm. ^b^Determined by HPLC analysis. ^c^Determined by HPLC analysis after derivatization.

In all cases the increase in temperature resulted in a decrease in reaction time compared to the reaction at room temperature (Table 1). It should be noted that in all reactions the product was obtained with high enantioselectivity (98% or higher). Among the (*R*)-selective enzymes the best was ATA-025-IMB as it gave in 3 hours at 50 °C 99% yield with >99% ee. ATA-415-IMB and ATA-013-IMB were also sensitive to the temperature effect; however, complete conversion of **1** was reached with these enzymes at 50 °C in 24 and 28 hours, respectively. The decrease in the reaction time observed with ATA-P1-G05-IMB in the reactions carried out at 35 °C and at 50 °C with respect to those at room temperature is particularly significant. Nevertheless, the time necessary for complete conversion was higher than that observed for (*R*)-selective enzymes at the same temperature. From the obtained results of the investigation of the temperature effect in the transamination reaction of **1** it was concluded that TAs-IMB exhibited higher activities at higher temperatures without loss of efficiency and selectivity.

Having in hand the good results obtained both in terms of activity and enantioselectivity, in particular with ATA-025-IMB at 50 °C, optimization of the reaction regarding the recycling of the enzyme was studied. Indeed, one of the advantages of using immobilized enzymes is the easy recovery of the enzyme at the end of the reaction by simple filtration and its reuse in successive reactions.

**Figure 1:**
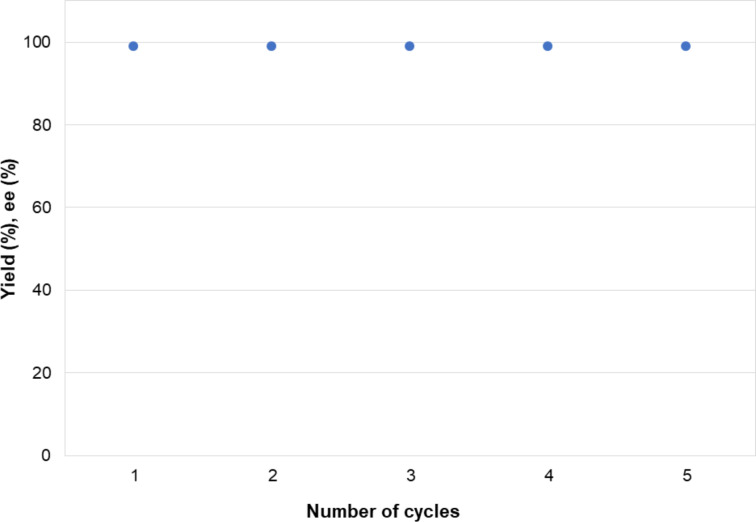
Reuse of ATA-025-IMB in five consecutive cycles in the transamination reaction of **1** in batch system (reaction time: 30 minutes in cycles 1, and 2 and 1 hour in cycles 3–5; temperature: 50 °C).

As previously indicated, in the transamination reaction it is necessary to use DMSO as a co-solvent to solubilize the substrate **1**, which is insoluble in an aqueous medium. In batch recycling reactions, it was tested if the reused enzyme would be partially deactivated after several reaction cycles in the presence of the organic solvent. Therefore, the amount of enzyme was increased in order to reduce the reaction time and as a consequence to limit the contact time of the enzyme with DMSO. The enzymatic transamination reaction was carried out as previously described at 50 °C but using 1 g of enzyme. At the end of the reaction, the enzyme was filtered, washed and reused in five consecutive reactions under the same experimental conditions (Figure 1).

Under these experimental conditions, complete conversion was obtained after 30 minutes of reaction in the first and in the second cycle. In the following reactions, complete conversion was reached after 1 hour. The enantiomeric excess of (*R*)-**2** was >99% in all cases. These results showed that ATA-025-IMB can be reused in subsequent runs without loss of selectivity and therefore looks promising for the development of a large-scale reaction. Furthermore, it should be noted that the selected enzyme allowed obtaining the (*R*)-enantiomer which is of greater industrial interest than the (*S*)-enantiomer.

**Figure 2:**
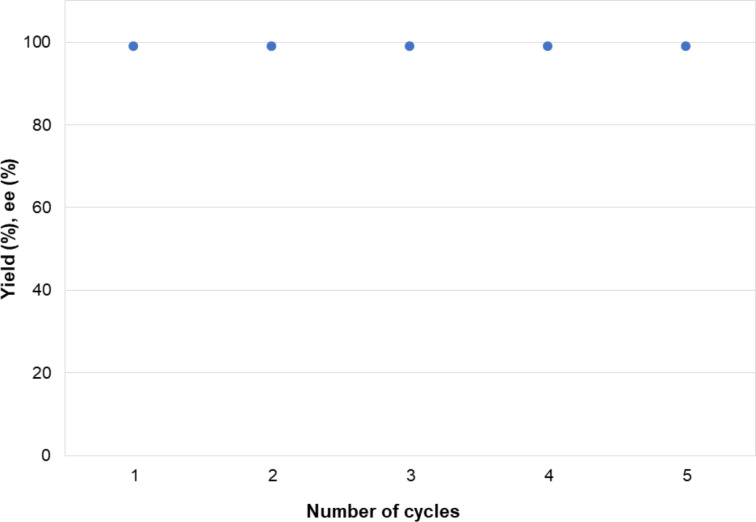
Reuse of ATA-025-IMB IMB in five consecutive cycles in the transamination reaction of **1** in a flow system (reaction time: 4 hours; temperature: 50 °C).

The good results obtained in the batch recycling reactions prompted us to investigate the possibility of a recycling process under flow conditions. For this purpose a microreactor was built consisting of a PEEK column which was filled with the enzyme. The reaction mixture was circulated within the reactor by using a suitable pump. Both the PEEK column and the reaction mixture were thermostated at a temperature of 50 °C. The progress of the reaction was monitored with the same methods used for the previously described reactions, by withdrawing aliquots of the reaction mixture. After 4 hours, when a complete conversion was achieved, the reaction was interrupted. The reaction mixture was then removed and the column was washed first with a 5% (v/v) aqueous solution of DMSO in H_2_O/triethanolamine buffer at pH 7.5 followed by H_2_O/triethanolamine buffer at pH 7.5 for the quantitative isolation of the reaction product. The column was then used in five consecutive reactions under the same experimental conditions. The results are shown in Figure 2.

In all cycles, the same values of conversion and enantiomeric excess as in the batch reactions were obtained. These results are of interest for application as they show that the activity of the enzyme was maintained even after several hours of continuous operation at 50 °C in the presence of DMSO. It is also noteworthy that when the reactor was stored at 4 °C and reused after several months under the same experimental conditions for further five runs on the substrate **1**, no relevant changes in activity and selectivity were obtained.

In order to optimize the reaction, some scale-up tests were performed using 200 mg of ATA-025-IMB and a substrate concentration of 40 g/L. Compared to the reactions carried out with smaller amounts of substrate, the reaction was slower which is most likely due to low solubility of the starting material under these experimental conditions. After 24 hours, complete conversion of **1** was obtained but in addition to the desired product (*R*)-**2** some byproducts were also observed, confirming possible degradation of the substrate in aqueous media [25]. However, (*R*)-**2** was obtained in 70% isolated yield and >99% ee. Finally, the product was converted into the corresponding dihydrochloride and compared with an authentic commercial sample in order to confirm the enantiomeric excess and absolute configuration.

## Conclusion

Several (*S*)- and (*R*)-selective immobilized transaminases have been investigated for the synthesis of a high added value chiral amine from the corresponding commercial ketone employing isopropylamine as amine donor. Due to the excellent enantioselectivity of the selected enzymes, the target molecule could be prepared with high yield and excellent enantiomeric excess. The stability of the enzyme with respect to the temperature and the organic solvent allowed its reuse both in batch and flow system.

## Experimental

### General

The chemicals used in this study were purchased from Sigma-Aldrich, Fluka or Alfa Aesar and used as received unless otherwise stated. An authentic sample of (*R*)-3-aminopiperidine dihydrochloride was obtained from Cangzhou Senary Chemicals S. & T. CO., LTD (China). Codex^®^ ATA-IMB Screening Kit was purchased from Purolite. The enzyme loading was about 100 mg protein per gram of wet resin. ^1^H and ^13^C NMR spectra were recorded at room temperature at the field indicated on a Bruker Avance II spectrometer. Multiplicities for ^1^H NMR couplings are shown as s (singlet), d (doublet), m (multiplet). Chemical shifts (in ppm) are referenced to residual protonated solvent. The optical rotations were measured on an Anton Paar MCP 300 polarimeter. Thin-layer chromatography (TLC) analyses were performed on silica gel 60 F254 plates using EtOAc/EtOH 1:1 as eluent and visualizing compounds by exposure to 0.2% ninhydrin solution and UV light or I_2_ vapor. The transamination reactions were performed in an Eppendorf ThermoMixer C which combines temperature control and mixing.

#### Chromatographic analysis

Analyses of the reactions were performed on a Jasco HPLC system with a Gemini C18 column (150 × 4.6 mm). Elution was carried out at 1 mL/min with detection at 220 nm and a column temperature of 25 °C. The eluent was H_2_O/CH_3_CN/DEA 70:30:0.1. Authentic standards were analyzed before analysis of the reaction mixtures.

The enantiomeric excesses were determined after derivatization. An aliquot (50 mg) of the crude sample and 100 mg K_2_CO_3_ were added to a 15 mL volumetric flask and dissolved in 1 mL of CH_3_CN and 4 mL H_2_O. Benzyl chloroformate (100 μL) was slowly added dropwise and the solution was sonicated for 5 minutes. After the addition of 1 mL ethyl acetate the solution was sonicated for further 5 minutes and extracted with ethyl acetate (1 mL). The organic phase was dried over Na_2_SO_4_, filtered and after evaporation of the solvent diluted with the mobile phase. Enantiomer separation was achieved on a Jasco HPLC system and a Lux-Cellulose 3 (250 × 4.6 mm) column with a flow rate of 0.5 mL/min, detection at 216 nm and column temperature of 25 °C. The eluent was hexane/isopropanol 9:1. The racemic compound was used as reference. The absolute configuration was assigned by comparison of elution order of an authentic standard.

#### General procedure for the transamination reaction with immobilized enzymes

In a similar manner as previously described in [14], to 5 mL of a triethanolamine buffer (100 mM, pH 7.5) containing isopropylamine (1.1 M), TA-IMB enzyme (200 mg) and PLP (1.4 mM) were added. The mixture was stirred at 35 °C and 550 rpm for 5 minutes, and then a preheated solution (35 °C) of 1-Boc-3-piperidone (**1**, 0.26 mmol, 45 mM) in DMSO (750 μL, 13% v/v) was added. The reaction was stirred at 35 °C and 550 rpm in an open vessel for 24 hours. The reaction was monitored by HPLC analysis and by TLC. At the end of the reaction, the enzyme was filtered under vacuum and washed with triethanolamine buffer (100 mM, pH 7.5, 3 × 2 mL). The recovered enzyme was suspended in buffer and stored at 4 °C. HCl (4 M) was added to the reaction mixture to reach pH 2 and the aqueous layer was extracted with CH_2_Cl_2_ (2 × 5 mL) to remove any starting material. After that, the pH of the solution was changed to pH 13 by addition of KOH and the aqueous layer extracted with CH_2_Cl_2_ (4 × 5 mL). The combined organic extracts were dried over Na_2_SO_4_, filtered and then evaporated under reduced pressure to yield 3-amino-1-Boc-piperidine (**2**). Each of the crude reaction mixtures were analyzed by NMR and HPLC.

#### Reuse of immobilized TA in batch system

The transamination reaction of 1-Boc-3-piperidone (**1**) was conducted using 1 g of ATA-025-IMB under the experimental conditions described above. Upon complete conversion, the enzyme was filtered, washed with triethanolamine buffer (100 mM, pH 7.5) and reused under the same reaction conditions for five subsequent reactions. The reaction mixtures were extracted and analyzed as described above.

#### General procedure for the transamination reaction with immobilized TA in flow system

ATA-025-IMB (0.8 g) was packed into a PEEK column which was then connected to a suitable pump. Triethanolamine buffer (100 mM, pH 7.5) containing isopropylamine (1.1 M) and PLP (1.4 mM) was circulated through the column at 35 °C, at a flow rate of 4 mL/min prior to use. Then a preheated solution of 1-Boc-3-piperidone (**1**, 0.26 mmol, 45 mM) in DMSO (750 μL, 13% v/v) was added. The reaction mixture contained in an open vessel was pumped through the column at 50 °C. The reaction was monitored by HPLC analysis and TLC. Upon complete conversion, the column was washed with triethanolamine buffer (100 mM, pH 7.5) to quantitatively recover the product. The column was stored at 4 °C. The eluted solution and washings were extracted as described above. The column was reused in five consecutive reactions under the same experimental conditions.

#### Preparative scale transamination reaction with immobilized TA

To 4 mL of triethanolamine buffer (100 M, pH 7.5) containing isopropylamine (1.1 M) and PLP (1.4 mM) ATA-025-IMB enzyme (200 mg) was added. The mixture was stirred at 35 °C and 550 rpm for 5 minutes, and then a preheated solution of 1-Boc-3-piperidone (**1**, 1.25 mmol, 208 mM) in DMSO (2 mL, 33% v/v) was added. The reaction mixture was stirred at 50 °C, 550 rpm and monitored by HPLC analysis and TLC. Upon complete conversion, the enzyme was filtered under vacuum and the reaction mixture was worked up as described above. After removal of the solvent of the combined organic phases under reduced pressure, (*R*)-**2** was obtained as a yellow oil with 70% isolated yield and >99% ee. ^1^H NMR (400 MHz, CDCl_3_) δ 3.91 (bs, 1H), 3.81 (d, 1H), 2.81 (ddd, 1H), 2.78 (m, 1H), 2.57 (bs, 1H), 1.89 (m, 1H), 1.66 (m, 1H), 1.45 (m, 1H), 1.44 (s, 9H, (CH_3_)_3_), 1.23 (m, 1H); ^13^C NMR (101 MHz, CDCl_3_) δ 155, 79.4, 52.12, 47.6, 43.64, 33.96, 28.43, 23.74; [α]_D_^20^ = −25.63 (*c* 0.31, EtOH) for the (*R*)-enantiomer, lit. [28]: [α]_D_^20^ = +26.0 (*c* 0.308, EtOH) for the (*S*)-enantiomer.

#### Synthesis of (*R*)-3-aminopiperidine dihydrochloride

In a vial, 100 mg (0.5 mmol) of (*R*)-1-Boc-3-aminopiperidine ((*R*)-**2**, ee >99%) were dissolved in 100 μL EtOH. To the resulting solution, 400 μL of a solution of acetyl chloride (2.8 mmol) in EtOH (1:1) was gradually added at 0 °C. The reaction mixture was then stirred at room temperature. A white solid settled down and indicated the completion of the reaction. The supernatant was decanted and the solid was washed with cold ethanol (2 × 100 μL). The solid was dried under vacuum to yield (*R*)-3-aminopiperidine dihydrochloride. ^1^H NMR (400 MHz, D_2_O) δ 3.73 (m, 2H), 3.47–3.44 (m, 1H), 3.06–2.89 (m, 2H), 2.19–2.17 (m, 1H), 2.03–2.00 (m, 1H), 1.76–1.73 (m, 1H), 1.61–1.64 (m, 1H); ^13^C NMR (101 MHz, D_2_O) δ 45.61, 44.94, 43.41, 26.26, 20.15; [α]_D_^20^ = −0.74 (*c* 0.5, CH_3_OH), lit. [22]: [α]_D_^20^ = −0.80 (*c* 0.5, CH_3_OH) for the (*R*)-enantiomer.
